# Genomic Signatures of Polyploid Origin and Diploidization in *Campanula americana*

**DOI:** 10.1093/gbe/evag252

**Published:** 2026-10-14

**Authors:** Alfredo López-Caamal, Laura F Galloway, Karen B Barnard-Kubow

**Affiliations:** Department of Biology, University of Virginia, Charlottesville, VA, USA; Department of Biology, University of Virginia, Charlottesville, VA, USA; Department of Biology, James Madison University, Harrisonburg, VA, USA

**Keywords:** Autopolyploidy, Whole-genome duplication, Comparative genomics, Genome evolution, Cytological diploidization, Genic diploidization

## Abstract

Cycles of whole-genome duplication (WGD) followed by return to diploidy have played a major role in plant evolution, yet genome evolution during intermediate stages of diploidization in autopolyploids remains less well characterized than in allopolyploids. We present a chromosome-scale genome assembly of *Campanula americana* to reconstruct its polyploid history and examine post-WGD diploidization. Our analyses revealed three WGDs, the most recent occurring approximately 10.4 million years ago. Multiple lines of evidence support an autopolyploid origin for *C. americana*, with none showing the chromosome-scale differentiation expected under allopolyploidy. At the structural level, duplicated homologous regions show extensive inversions and translocations, and the current chromosome number is reduced relative to the ancestral base number. These patterns are consistent with substantial cytological diploidization. In contrast, genic diploidization appears more gradual and balanced. Gene retention is broadly symmetric between homologous regions, and losses occur primarily as dispersed single-gene events rather than block-scale deletions, with no evidence for subgenome dominance. This pattern is consistent with limited inherited asymmetries between duplicated regions and differs from allopolyploids that show subgenome-biased fractionation. Phylogenetic and cytological evidence further suggest that the most recent WGD is shared across a small North American clade. Together, our results characterize *C. americana* as a polyploid in an intermediate diploidization stage (ie mesopolyploid), in which chromosome-level reorganization appears more advanced than gene fractionation. This genome establishes a foundation for comparative studies of genome evolution following WGD across the North American Campanulaceae and more broadly for understanding how polyploid genomes return to a diploid-like state.

Significance Repeated cycles of polyploidy have played a central role in plant diversification, yet how polyploid genomes transition back to a diploid state, particularly the intermediate stages, remains poorly understood. The chromosome-scale genome of tetraploid *Campanula americana* provides a snapshot of this process. At the structural level, extensive chromosomal reorganization among duplicated regions is consistent with cytological diploidization since the most recent whole-genome duplication. At the genic level, diploidization is ongoing, proceeding in a gradual, balanced manner, with gene losses dispersed across homologous regions and no evidence for subgenome dominance. Accordingly, genome-wide analyses find none of the subgenome differentiation expected if the duplicated genomes came from divergent parental lineages, pointing to an autopolyploid rather than an allopolyploid origin. These findings show that cytological and genic diploidization can progress at different rates, with chromosome-level diploidization advancing more rapidly than gene-level fractionation. Our results place *C. americana* as a promising model for understanding how polyploid genomes revert to a diploid-like state.

## Introduction

Whole-genome duplication (WGD), or polyploidy, is a pervasive feature of plant evolution (Parisod et al. 2010; Van de Peer et al. 2017). Nearly all angiosperms show evidence of at least one ancient WGD, and many lineages have experienced multiple independent events. Polyploidy arises in two primary ways: autopolyploidy, through genome duplication within a single species, and allopolyploidy, through genome duplication following hybridization between divergent species (Van de Peer et al. 2017). Both mechanisms instantly double the nuclear genetic material, providing a vast amount of raw material on which selection can act. This redundancy allows for gene neo- and subfunctionalization, enabling the evolution of novel metabolites, enhanced stress tolerance, and increased ecological adaptability (Mandáková et al. 2017; Xiao et al. 2020; Feng et al. 2024; Salmon et al. 2025). As a result, WGD has profoundly influenced plant diversification from the earliest angiosperms to modern crops, leaving a lasting imprint on their genomes (Jiao et al. 2011; Renny-Byfield and Wendel 2014). Understanding how genomes evolve following WGD is therefore central to explaining contemporary plant diversity (Spoelhof et al. 2017). This requires elucidating the processes and patterns that restructure polyploid genomes over time, such as gene loss, subgenome differentiation, and diploidization, which ultimately determine how duplicated lineages stabilize, diversify, and give rise to the variation observed among plant species today.

Polyploids frequently undergo diploidization, a process that restores diploid-like chromosome pairing and gene content (Tayalé and Parisod 2013; Mandáková and Lysak 2018; Li et al. 2021). Diploidization operates through two major mechanisms that act on different timescales: cytological diploidization and genic diploidization (or fractionation) (Ma and Gustafson 2005; Renny-Byfield and Wendel 2014). The trajectory of diploidization differs between auto- and allopolyploids, reflecting their distinct genomic origins and influencing how chromosomal pairing stabilizes and how gene redundancy is resolved following WGD (Soares et al. 2021; Gonzalo 2022).

In newly formed polyploids, changes in meiotic behavior are critical for stabilizing inheritance (Yant et al. 2013; Gonzalo 2022). In allopolyploids, divergence between chromosomes of the distinct parental taxa naturally promotes preferential pairing and facilitates the establishment of disomic inheritance with limited cytological modification (Ma and Gustafson 2005). In contrast, in autopolyploids, where homologous chromosomes are nearly identical, meiosis is instead characterized by multivalent formation and polysomic inheritance (Parisod et al. 2010; Soares et al. 2021). Even when bivalent pairing occurs, it does not ensure disomic inheritance because pairing homologs can remain random (Lv et al. 2024). Cytological diploidization in autopolyploids therefore requires the progressive elimination of random pairing and segregation, a process that may proceed gradually and can remain incomplete over evolutionary timescales (Gundappa et al. 2022). Structural rearrangements, such as fusions, fissions, or translocations, contribute to this process, but their role and tempo vary among taxa (Parisod et al. 2010; Li et al. 2021; Deb et al. 2023).

In parallel, genic diploidization gradually reshapes the genome by removing or modifying duplicate genes through pseudogenization, deletion, or neofunctionalization (Freeling et al. 2015; Li et al. 2021). In many allopolyploids, this process is asymmetric, showing biased fractionation in which one parental genome, or subgenome, is preferentially retained and expressed (Bird et al. 2018; Cheng et al. 2018). The resulting pattern, known as subgenome dominance, has been well documented in crops such as corn (Schnable et al. 2011), cotton (Yoo et al. 2013), and wheat (Harper et al. 2016). These biases often coincide with gene losses across large contiguous genomic regions (ie block-scale gene losses) and are thought to be driven by differences in transposable element composition and epigenetic state inherited from divergent parental genomes (Zhao et al. 2017; Bird et al. 2018; Cheng et al. 2018). By contrast, autopolyploids arise within a single lineage, so their duplicated genomes are nearly identical and therefore expected to lack the inherited epigenetic asymmetries that drive subgenome asymmetries typical of allopolyploids (Liu and Wang 2023). Yet, subgenome-like dominance has been reported in some autopolyploid species (Yu et al. 2023). In total, the processes and tempo of genic diploidization in autopolyploids, particularly during intermediate stages of genome evolution, remain less well understood.

Although these contrasting trajectories of diploidization have been well established conceptually, how these processes progress during intermediate stages following autopolyploidy remains less characterized (Parisod et al. 2010; Soltis et al. 2010, 2016; Tayalé and Parisod 2013; Garsmeur et al. 2014; Spoelhof et al. 2017; Baduel et al. 2018). Studies of ancient autopolyploid genomes show that duplicated regions can retain largely symmetric patterns of divergence and fractionation over long evolutionary timescales (Garsmeur et al. 2014), while most of our understanding of the earlier stages comes from recent autopolyploid crops or model species such as potato (Sun et al. 2025) and *Arabidopsis* (Gonzalo et al. 2023; Parra-Nunez et al. 2024). In contrast, studies on natural autopolyploids at intermediate stages of diploidization remain limited. Therefore, additional genomes from such species are needed to characterize how gene retention and fractionation occur as genomes transition back to a diploid-like state.

*Campanula americana* L. (=*Campanulastrum americanum* (L.) Small), a monocarpic herb native to eastern North America, offers an opportunity to address this gap. This species belongs to the Campanulaceae sensu *lato*, a family marked by multiple independent WGD events (Crowl et al. 2016; Xu et al. 2025), but with limited genomic resources. Cytological studies show that while the subclade Campanuloideae typically has *n* = 17 chromosomes, *C. americana* has *n* = 29 across its range (Gadella 1964; Galloway et al. 2003; Crowl et al. 2016), consistent with a WGD followed by chromosome loss and/or structural reorganization. Allozyme segregation patterns have been interpreted as evidence for tetrasomic inheritance, suggesting an autopolyploid origin (Galloway et al. 2003). However, the origin of this polyploid remains uncertain, as similar patterns could arise under different scenarios such as in a diploidized allopolyploid or from methodological limitations of earlier approaches. Thus, *C. americana* represents a useful system to investigate the genomic consequences of WGD and reconstruct the origin and evolutionary history of its polyploidization.

Here, we present a chromosome-scale assembly of *C. americana* generated using PacBio HiFi and chromatin conformation capture (Hi–C) sequencing. We reconstruct its WGD history using three complementary approaches: synonymous substitution (*Ks*) distributions, synteny depth analyses, and phylogenetic reconciliation. Our results reveal that *C. americana* underwent three separate WGDs. We provide phylogenetic evidence that the most recent WGD event is shared by a small North American clade comprising *C. americana*, *Triodanis*, and related species. Genome-wide analysis, including k-mer subgenome analysis, LTR-RT insertion age profiles, and chromosome–pair Ks distributions, found none of the subgenome differentiation expected under allopolyploidy, supporting an autopolyploid origin for the most recent WGD. Genic diploidization appears to have proceeded in a gradual, balanced manner with no evidence of subgenome dominance, whereas substantial structural reorganization indicates more advanced chromosome-level diploidization. Together, these findings characterize *C. americana* as a mesopolyploid at an intermediate stage of diploidization and provide a useful system to investigate how structural and genic diploidization progress following WGD.

## Results

### Genome Assembly and Annotation of the *Campanula americana* Genome

We assembled the *C. americana* genome using 82.16 Gb of PacBio HiFi sequencing data and 691.7 million Hi–C reads (Table S1). An initial assembly using only PacBio HiFi sequencing data produced a 3.25 Gbp assembly in 8,380 contigs. This size is nearly twice the k-mer based estimate (1.65 Gbp; see *Genome profiling*), suggesting that the initial assembly contained two haplotypes. Incorporating Hi–C data allowed chromosome-scale scaffolding, resulting in 58 pseudochromosomes comprising 92% of the assembly (Table S2). The number of pseudochromosomes agrees with cytological counts in this species (Gadella 1964).

Reciprocal alignment of scaffolds allowed the identification of homologous chromosome pairs, enabling separation of the 58 pseudochromosomes into two haplotypes. Within each homologous pair, the longest and more complete pseudochromosome was assigned to Haplotype 1, while the other was assigned to Haplotype 2 (Table S3). For downstream analyses, we used Haplotype 1, comprising 29 pseudochromosomes (hereafter referred simply as chromosomes) with a total length of 1.69 Gbp and a scaffold N50 of 50 Mbp. The Hi–C contact map of Haplotype 1 showed a strong intrachromosomal interaction signal along the diagonal and limited off-diagonal contacts, supporting correct scaffold ordering and orientation (Fig. S1).

BUSCO analysis estimated the genome completeness to be as high as 98.5% (Table S4), with 72.7% of genes found to be duplicated, as expected for a polyploid.

We predicted 45,735 protein coding genes using a combination of ab initio, homology-based, and transcriptome-based predictions. BUSCO analysis of the predicted gene set confirmed high completeness with 97.7% of genes in the embryophyta_odb12 database recovered. Repetitive element annotation revealed that 1.49 Gbp (75.7%) of the genome is composed of transposable element repeats (Fig. 1c). Long Terminal Repeats retrotransposons (LTR-RT) accounted for 44.1% of the genome, with *Gypsy* and *Copia* elements the most abundant classes (Table S5). An additional 26.6% of repeats could not be classified. In all, these results show the high continuity and quality of the genome assembly of *C. americana*.

**Fig. 1.**
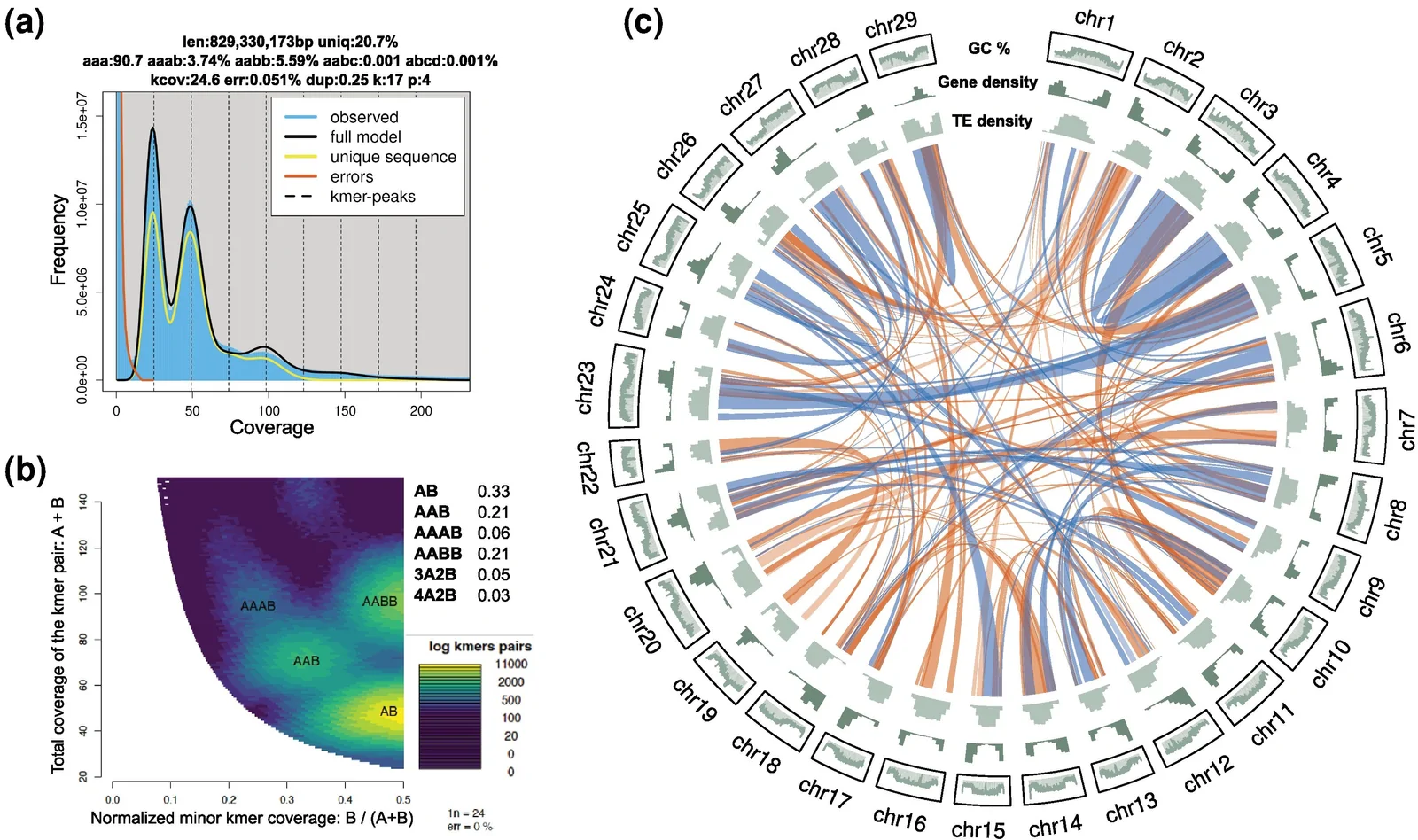
Genome profiling and assembly of the autopolyploid *Campanula americana*. a) GenomeScope k-mer profile distribution based on 17-mers. Peaks correspond to different copy number classes, indicating high level of homozygosity and supporting a tetraploid genome structure. b) Smudgeplot analysis based on k-mer coverage ratios, revealing the genome structure typical of an autotetraploid with prevalent AABB and AAB configurations. c) Circular representation of the assembled *C. americana* genome showing extensive reorganization. Concentric tracks (from outer to inner): GC content, gene density, and transposable element density. Central links represent syntenic blocks, with orange blocks indicating an old, lineage-specific whole-genome duplication (WGD) event and blue synteny from the most recent WGD.

### Genome Profiling

Genome profiling based on both *k*-mer distribution (GenomeScope) and k-mer pair co-occurrences (Smudgeplot) strongly suggest tetraploidy (Fig. 1). The presence of a prominent peak at ∼25×, along with smaller peaks at ∼50×, 75×, and 100×, indicates a tetraploid genome with high levels of homozygosity (Fig. 1a). Additionally, the Smudgeplot analysis reveals a stoichiometry consistent with autopolyploidy, with the main allelic dosage proportions being AB = 0.33, AAB = 0.21, and AABB = 0.21 (Fig. 1b). In contrast, finding only AB and AABB would reflect the presence of two distinct subgenomes expected for allopolyploids. Genome size was estimated at 1.65 Gbp, which is reasonably close to both the size of our assembly (1.69 Gbp) and to previously reported flow cytometry estimates (∼2 Gbp; Galloway et al. 2003).

### Evidence for Three WGDs in *Campanula americana*

All three approaches used for WGD inference (Ks distributions, synteny depth, and phylogenetic reconciliation) support three events in the evolutionary history of *C. americana* (Fig. 2). Based on *Ks* distribution analyses of paralogs, the oldest duplication occurred approximately 145 Mya. This event was also present in other Campanulaceae species, including *C. pilosula*, *L. dortmanna*, and *L. seguinii*, suggesting it corresponds to the core eudicot γ-WGT event (Fig. 2a). In addition, all approaches identified two, more recent lineage-specific WGDs in *C. americana*. Ks-based dating places one at ∼35 Mya, shared with other *Campanula* and *Adenophora* species, and the most recent at ∼10.4 Mya. The Ks distribution of orthologs, which reflect divergence times, supports this interpretation, as the estimated duplications occurred after divergence of related species (Fig. 2a, c).

**Fig. 2.**
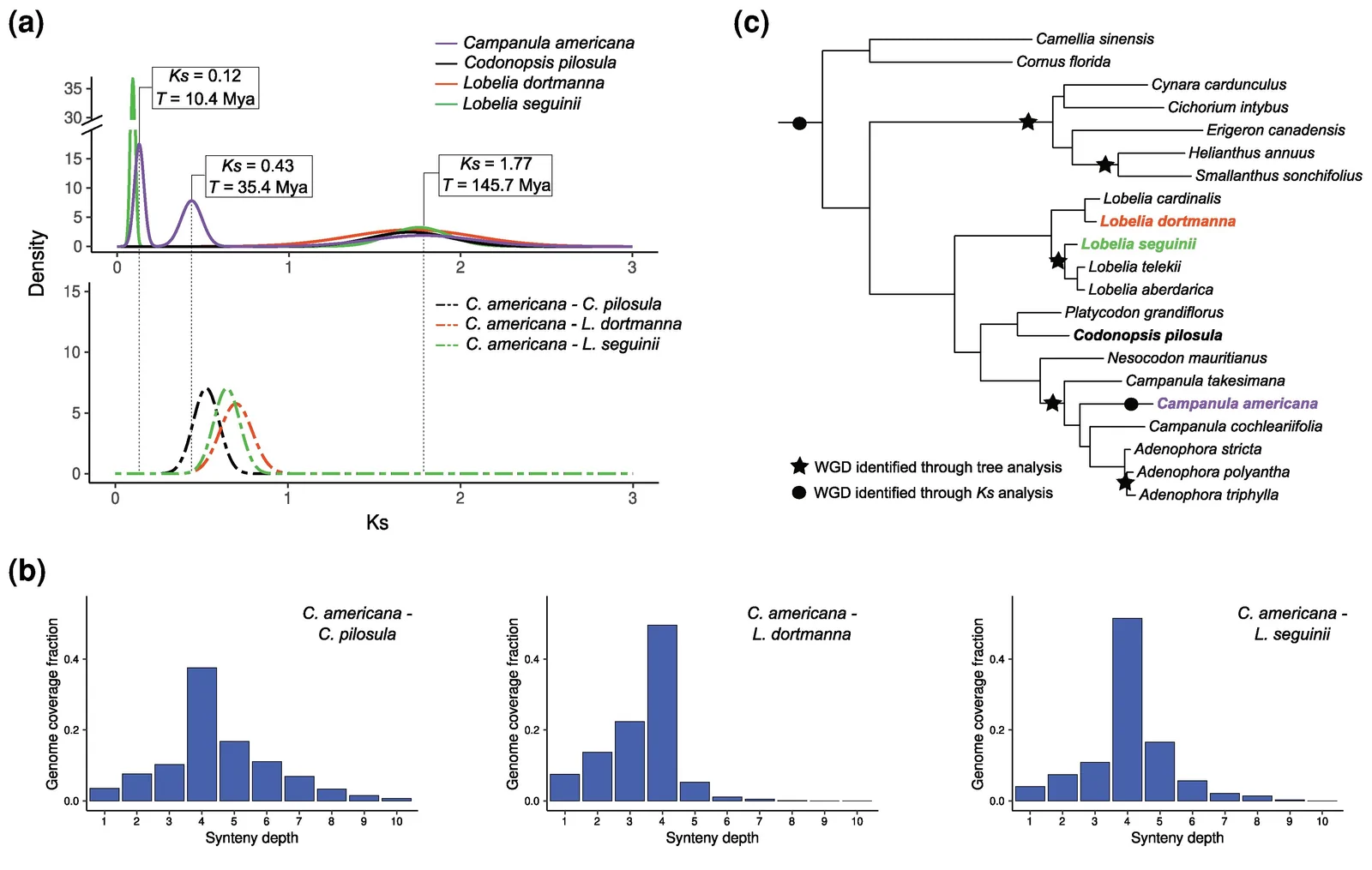
Whole-genome duplication evidence for *Campanula americana*. a) Synonymous substitution rate (*Ks*) distribution reveals peaks corresponding to ancient and recent WGDs across *C. americana* and related species. There is one recent Ks peak (*Ks* = 0.12, ∼10.4 Mya) and two older peaks (*Ks* = 0.43 and 1.77) reflecting more ancient WGDs. Lower panel shows orthologous *Ks* distributions between *C. americana* and *Codonopsis pilosula*, *Lobelia dortmanna*, and *L. seguinii*, reinforcing divergence timings. b) Synteny depth between *C. americana* and other members of Campanulaceae. All species show a modal depth of four, which suggests two WGDs since their divergence from *C. americana*. c) Phylogenetic placement of WGD events using both tree-based (star) and *Ks*-based (circle) inference methods. Two independent, lineage-specific WGD events are identified within the *C. americana* lineage.

Synteny depth analyses further support two WGDs in *C. americana* after divergence from other Campanulaceae (*C. pilosula* and *Lobelia* spp.). The projection of *C. americana* collinear blocks onto these species yielded a modal syntenic depth of 4, consistent with two rounds of duplication (Fig. 2b). The corresponding duplicated regions were easily identifiable in the Ks dotplot. Most blocks associated with the most recent WGD (*Ks* = 0.05 to 0.25) showed extensive chromosomal rearrangements including inversions and translocations; however, some chromosomes, such as chr3 and chr4, showed markedly fewer rearrangements. On the other hand, blocks associated with the older lineage-specific duplication (*Ks* = 0.3 to 0.6) persist but are more fragmented, consistent with progressive rearrangement and fractionation (Fig. 1c).

Broad-scale phylogenetic reconciliation analysis was consistent with these results and also recovered previously reported WGD events in other clades, including a duplication common to the Asteraceae and one within the Lobelioideae (Fig. 2c). However, some *Lobelia* species, such as *L. dortmanna* and *L. cardinalis*, did not show evidence of clade-specific WGD events. For example, *L. dortmanna* displayed only a single peak in its Ks distribution, consistent with the absence of recent duplication (Fig. 2a). The ASTRAL species tree was strongly supported, with all nodes receiving LPP = 1, providing strong confidence of the inferred relationships (Fig. 2c).

To refine the phylogenetic placement of the most recent WGD, we conducted a second reconciliation analysis using a denser sampling of closer relatives of *C. americana*. The species tree, inferred from 510 single-copy genes, recovered the European genus *Legousia* as sister to a North American clade comprised of several *Campanula* species, including *C. americana*, and the *Triodanis* species (hereafter *Campanula–Triodanis* clade; Fig. S2). Tree2GD identified elevated duplication signals on two adjacent branches: the branch ancestral to *Legousia* + *Campanula–Triodanis* (8 inferred duplications among 79 evaluated gene tree nodes, GDratio = 10.13%; Fig. S2) and the branch subtending the *Campanula–Triodanis* clade (15/171, GDratio = 8.77%; Fig. S2). Because these alternative placements differ in whether the WGD preceded or followed divergence of *Legousia*, we examined the gene trees contributing to each signal. If the WGD occurred before the divergence of *Legousia*, duplicated *Legousia* copies would be expected to occur in both paralog lineages. However, none of the supporting gene trees showed this pattern. Instead, *Legousia* was represented only in one paralog lineage. In contrast, among the 15 gene trees supporting a duplication after the divergence from *Legousia*, 12 contained at least one *Legousia* species, and ten of these placed it outside both duplicated *Campanula–Triodanis* lineages (Fig. 3 lower panel). These results suggest that the most recent WGD occurred after the divergence of *Legousia* and before the diversification of the *Campanula–Triodanis* clade.

**Fig. 3.**
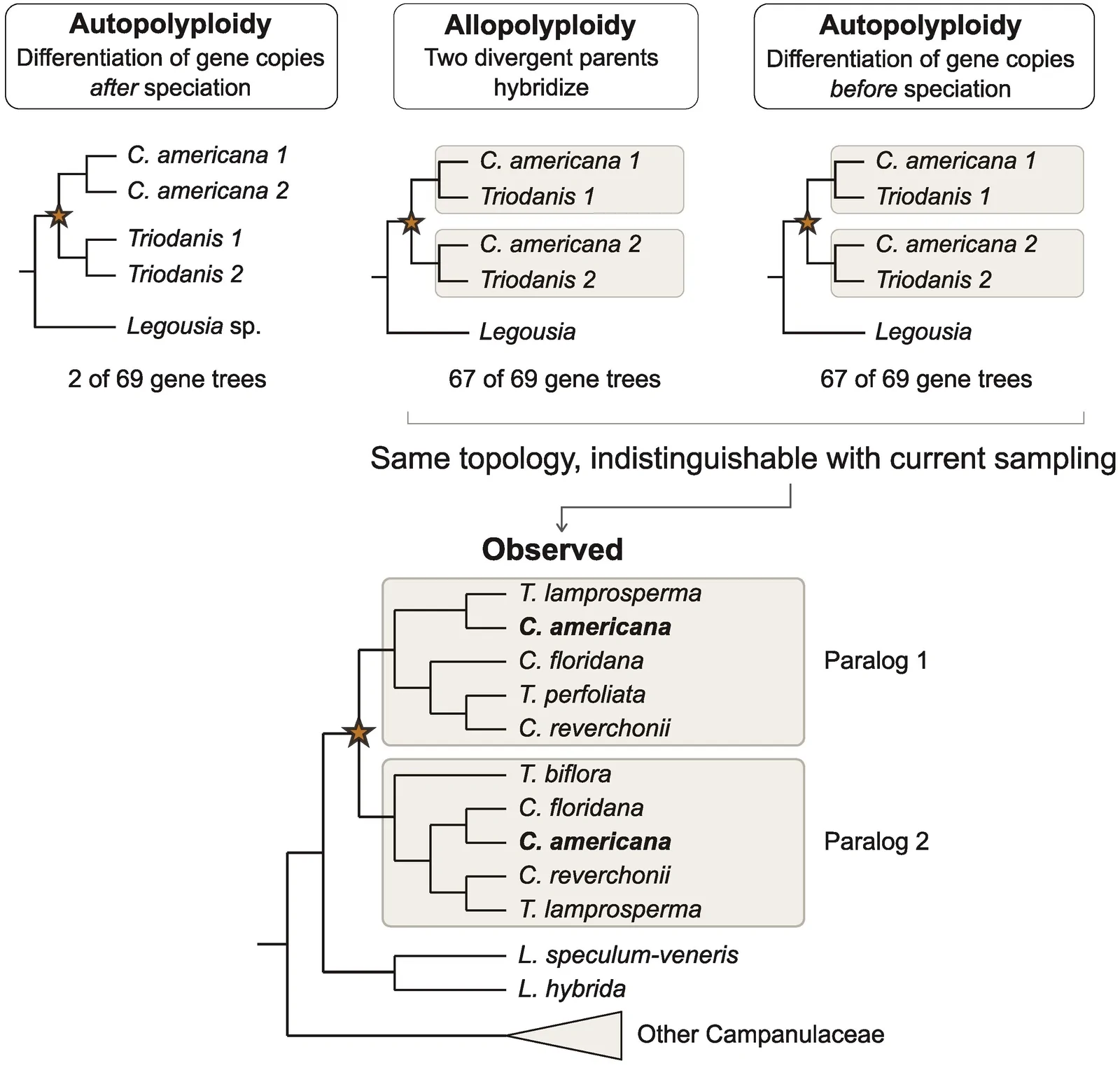
WGD scenarios and representative gene tree supporting the placement of the most recent *Campanula americana* WGD after the divergence of *Legousia*. Upper panels show the gene tree topologies expected under each scenario, with the star indicating the WGD. Autopolyploidy, in which gene copies diverged only after the *Campanula–Triodanis* split, predicts that the two *C. americana* copies will be each other's closest relatives. This pattern was recovered in 2 of 69 gene trees. Allopolyploidy (central panel) and autopolyploidy in which copies differentiated before the clade radiated (right panel) both predict the two copies to fall into separate paralog lineages, each grouping with copies from the other *Campanula–Triodanis* taxa. This pattern was recovered in 67 out of 69 gene trees. The lower panel shows a representative gene tree (locus AT5G55960, encoding a TqsA-like transmembrane protein; the two *C. americana* copies are located in chr1 and chr26). The tree shows two *Campanula–Triodanis* paralog lineages, consistent with either of the latter two scenarios.

### Phylogenetic and Genomic Evidence Suggest an Autopolyploid Origin of *C. americana*

To examine the mode of the most recent WGD, we first explored the placement of the *C. americana* copies in the gene trees. Among the 69 trees containing more than one *C. americana* copy, only two (2.9%) recovered the copies as each other's closest relatives. This pattern is expected if copies differentiated after the *Campanula–Triodanis* lineages diverged. On the other hand, in 67 (97.1%) of the gene trees, each copy grouped with copies from other *Campanula–Triodanis* species, placing them in two different paralog lineages (Fig. 3). This topology is expected under allopolyploidy but also in autopolyploidy in which gene copies diverged before the radiation of the clade (Fig. 3 upper panel).

Although gene tree topology cannot distinguish between these scenarios in general, it can test one form of allopolyploidy. If the two parental lineages were distantly related, each *C. americana* copy should be most closely related to a different external lineage. Therefore, we identified the closest external lineage of each copy. Among the 29 gene families in which the external phylogenetic relationships of duplicated copies could be resolved (bootstrap ≥ 50 and both outgroup species monophyletic), 27 (93.1%) associated both copies with the same external lineage. In 25 of these 27 families, both copies showed their closest external relationship to *Legousia*. This excludes an allopolyploid origin involving parents from two distantly related lineages, but not one involving closely related parents that diverged after the split from *Legousia*.

To evaluate the remaining possibility of allopolyploidy between closely related species, we examined the *C. americana* genome for signatures of subgenome differentiation, which are expected if the parental lineages evolved independently before hybridizing. We did not identify chromosome-scale partitioning consistent with two differentiated parental subgenomes in *C. americana* (Fig. 4). Across all tested minimum k-mer frequency thresholds in SubPhaser (-*q* = 50, 25, 17, and 12; Jia et al. 2022), results were qualitatively consistent, with no separation of chromosomes into two balanced groups (only results for *q* = 25 are shown). Analyses based on a subset of chromosomes yielded weak subgenome assignments affecting only chromosomes 10 and 12 (Fig. 4a). Hierarchical clustering of differential k-mer abundance did not recover two equal chromosome sets but instead revealed largely homogeneous genomic patterns (Fig. 4b). Consistently, the density distribution of LTR-RT insertion ages showed largely overlapping confidence intervals between the two inferred groups, rather than two distinct TE landscapes (Group1: median = 0.23 Mya, 95% CI: 0 to 3.16; Group2: median = 0.36 Mya, 95% CI: 0 to 3.07).

**Fig. 4.**
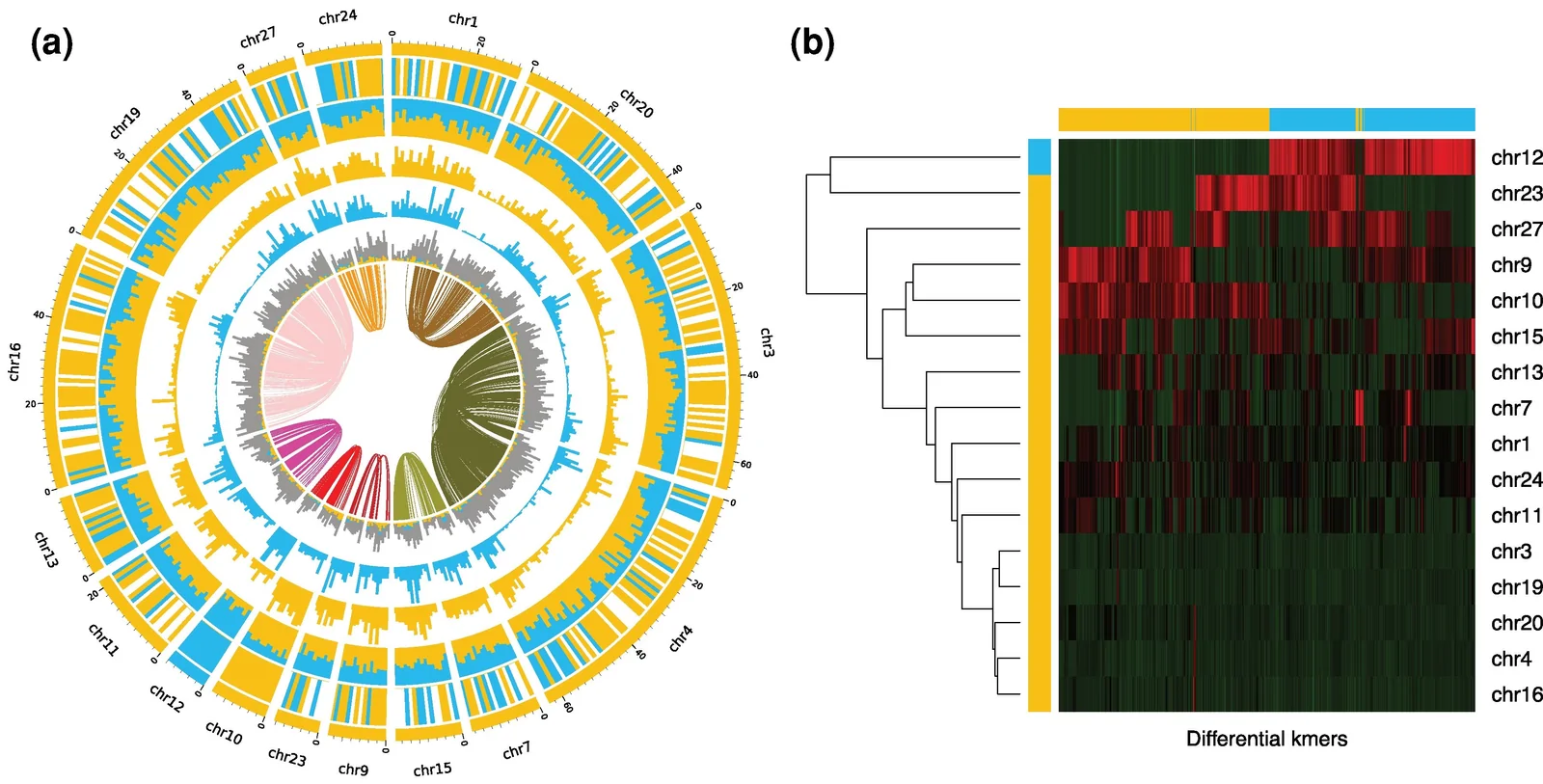
Lack of subgenome differentiation in *Campanula americana*. a) From outer to inner circles: (i) subgenome assignments based on k-means algorithm; (ii) enrichment of subgenome-specific k-mers, white areas denote no significant enrichment; (iii) relative proportion and (iv to vi) absolute count of subgenome-specific k-mers; (vii) density of LTR-RTs—gray indicates non-subgenome-specific LTR-RTs; (viii) homologous blocks. b) Heatmap and unsupervised hierarchical clustering analysis of differential k-mers. The horizontal bar at the top indicates the subgenome to which each k-mer is specific, while the vertical bar to the left indicates the subgenome assignment of each chromosome.

Finally, to further evaluate whether divergence patterns varied across the genome, we examined pairwise Ks among collinear gene pairs identified with WGDI, grouped by chromosome pairs. Ks distributions were highly similar across all chromosome combinations, with similar medians and no evidence of distinct chromosome classes (Fig. S3). Together with the absence of consistent alternative parental affinities in the gene trees, and the lack of chromosome-scale subgenome differentiation, these results support an autopolyploid origin of the most recent WGD in *C. americana*.

### Post-WGD Gene Retention and Lack of Subgenome Dominance

To test for subgenome-like dominance following the most recent WGD, we used synteny between homologous regions to construct “consensus chromosomal blocks,” defined as the union of all genes within a given collinear block. We then estimated the proportion of retained genes for each chromosome across all blocks (see Materials and Methods for details). Because chromosomes within collinear blocks could not be assigned to different subgenomes, we quantified retention asymmetry as the absolute difference in retention between the two chromosomes forming each block (|Δ_retention_|).

We found that the genome-wide retention asymmetry was low (|Δ_retention_| = 0.11 ± 0.12), indicating that both members of a given collinear block retain similar proportion of genes following the most recent WGD. In addition, the distribution of |Δ_retention_| was strongly skewed toward low values, with most blocks showing limited asymmetry and relatively few showing pronounced differences in retention (Fig. 5b).

**Fig. 5.**
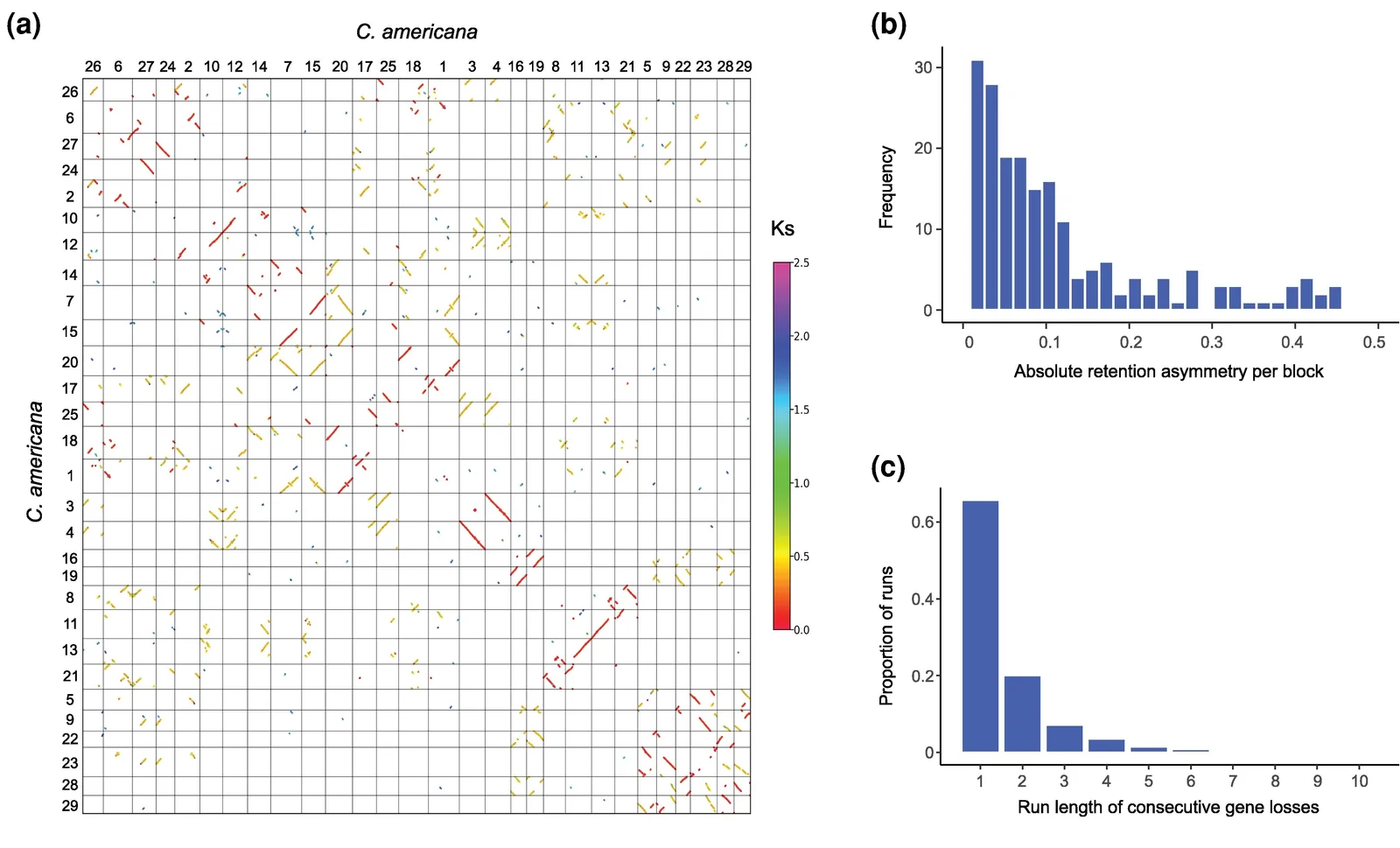
Analysis of whole-genome duplication (WGD) history and genome evolution in *Campanula americana*. a) Dotplot of collinear gene pairs within the *C. americana* genome. The colors represent different Ks values, with blue (*Ks* = 1.5 to 2.5) representing an ancient WGD event, orange (*Ks* = 0.3 to 0.65) an older lineage-specific WGD, and red (*Ks* = 0.02 to 0.25) the most recent WGD. b) Distribution of the absolute retention asymmetry (|Δ_retention_|) across all collinear blocks. Retention asymmetry was calculated as the absolute difference in the proportion of retained genes between homologous chromosomes. Values closer to zero indicate similar retention between homologs. The skewed distribution toward lower values indicates that most blocks exhibit modest differences in gene retention. c) Distribution of consecutive gene losses for each duplicated block relative to the consensus block. Most gene losses occur as short runs, indicating that gene loss is typically localized rather than occurring in large segments.

We next asked whether post-WGD gene losses occurred in clusters or as isolated events. For this purpose, we quantified the number of consecutive gene losses along all consensus chromosomal blocks. Frequency distributions revealed that nearly all losses occurred as single-gene events, with lower evidence for extended contiguous blocks of gene loss (Fig. 5c). This pattern indicates that gene loss after duplication was largely dispersed with a low frequency of block-scale events.

## Discussion

We generated a chromosome-scale genome assembly of *C. americana* to investigate the origin and evolution of its polyploid genome. Our results support a history of repeated genome duplication, including a recent WGD (∼10.4 Mya) in the *C. americana* lineage, and reveal a genome that retains strong tetraploid structure despite extensive chromosomal reorganization. To determine whether this polyploid arose through allopolyploidy, we applied multiple lines of evidence, none of which detected chromosome-scale differentiation that would be expected under an allopolyploid origin. Duplicated chromosomal regions show clear evidence of substantial post-WGD structural reorganization, including extensive translocations, and chromosome–number reduction. Genic diploidization (ie fractionation), by contrast, appears more gradual and balanced, with gene retention largely symmetric between homologous regions and gene loss occurring primarily as isolated events rather than block-scale deletions. Taken together, these findings support the tetraploid nature of *C. americana* and characterize its genome as consistent with an autopolyploid at an intermediate stage of diploidization (ie mesopolyploid). In addition, the *C. americana* genome provides a valuable resource for further investigating genome evolution across the Campanulaceae and for clarifying how repeated WGDs have shaped the history of the family.

### Genomic Evidence for the Origin of the *C. americana* Polyploid Genome

Distinguishing between autopolyploid and allopolyploid origins can be challenging, particularly in lineages that have undergone substantial diploidization. In recently formed allopolyploids, homeologous chromosomes are expected to retain the divergence inherited from their parental species, evidenced as chromosome-scale subgenome differentiation, distinct sequence divergence among homeologous regions, and contrasting transposable element landscapes (Schnable et al. 2011; Bird et al. 2018). However, these signatures may erode over time as a consequence of recombination, genome rearrangement, or biased gene loss (Renny-Byfield and Wendel 2014; Mandáková and Lysak 2018; Li et al. 2021). Therefore, the absence of clear subgenome structure cannot prove autopolyploidy, but it can be used to evaluate whether the genome retains the expected signatures of a differentiated allopolyploid origin.

In *C. americana*, we found no evidence for two differentiated parental subgenomes. Analyses failed to recover two balanced chromosome groups across a range of k-mer frequency thresholds. Hierarchical clustering of k-mer abundance similarly failed to partition the genome into two subgenome classes. The LTR-RT insertion age profiles were largely overlapping among inferred chromosome groups, rather than reflecting the distinct transposable element histories expected under allopolyploidy. In addition, pairwise *Ks* distributions among collinear block pairs were similar across all pairs, indicating that sequence divergence is relatively homogeneous across the genome rather than partitioned into divergent chromosome sets. Phylogenetic analyses are consistent with this interpretation but failed to unambiguously demonstrate an autopolyploid origin. The duplicated *C. americana* copies were most closely related to the same external lineage in nearly all gene trees, which excludes an allopolyploid origin from distantly related parents. However, the grouping of copies into two paralog lineages, each shared with other *Campanula–Triodanis* species, is expected under both allopolyploid and autopolyploid in which copies differentiated before the clade radiated. Closely related parental species that diverged shortly before hybridizing may also have left little detectable subgenome differentiation. Therefore, an allopolyploid of this kind cannot be excluded, and resolving it would require genomes from diploid relatives of the putative parental lineages, each grouping with only one of the two paralog lineages.

However, the genomic evidence does not support an origin from two differentiated parental genomes in *C. americana*. Instead, our results are consistent with an autopolyploid origin in which duplicated chromosomes arose within a single lineage and began with limited sequence and epigenomic divergence. This interpretation aligns with previous allozyme evidence for tetrasomic inheritance in *C. americana*. Therefore, we treat *C. americana* as a putative autopolyploid with ongoing diploidization while acknowledging that an ancient allopolyploid origin from closely related parental species remains possible.

### Patterns of Diploidization in the Autopolyploid *C. americana*

Diploidization involves changes at both chromosomal and genic levels. At the chromosomal level, diploidization refers to the restoration of diploid-like meiotic behavior, often through structural changes that reduce multivalent formation and promote preferential chromosome pairing (Lv et al. 2024). At the genic level, it involves the gradual elimination of duplicated gene content through gene loss, pseudogenization, or functional divergence (Ma and Gustafson 2005; Renny-Byfield and Wendel 2014; Freeling et al. 2015; Li et al. 2021). These two components of diploidization do not occur at the same rate, and as discussed below, *C. americana* illustrates this asymmetry.

At the structural level, synteny analyses reveal numerous inversions and translocations among homologous blocks in *C. americana*, indicating that duplicated chromosomes have not remained collinear since the most recent WGD. The current chromosome number (*n* = 29) is also lower than expected under a simple doubling of the ancestral Campanuloideae base number (*n* = 17; Crowl et al. 2016), indicating that chromosome fusions, losses, or other large-scale rearrangements have occurred since the last WGD event. Such structural changes are associated with diploidization and may facilitate preferential pairing and reduce the opportunity for multivalent formation (Parisod et al. 2010; Mandáková and Lysak 2018; Li et al. 2021; Gonzalo 2022). This dynamic is particularly relevant in an autopolyploid, where initially identical duplicated chromosomes may promote multivalent formation and delay diploid-like inheritance (Soares et al. 2021; Lv et al. 2024). Although hifiasm produced two partially phased assemblies, this is not necessarily evidence for completed cytological diploidization, as hifiasm is optimized for diploid organisms. Instead, evidence for chromosome-scale diploidization comes from the recovery of 29 Hi–C scaffolded pseudochromosomes, matching previous chromosome counts in the species (Gadella 1964).

In contrast, genic diploidization in *C. americana* appears more gradual and balanced. Our synteny analysis revealed that many duplicated regions associated with the most recent WGD remain identifiable, indicating that the genome has not returned to a diploid-like gene content. Retention asymmetry between homologous chromosome pairs was low across the genome, and gene losses occurred primarily as isolated single-gene events rather than extended block-scale deletions. These results indicate that fractionation has been neither preferentially directed toward one chromosome set nor dominated by large deletions. This stands in contrast to well-characterized allopolyploids such as maize and *Brassica*, where one subgenome consistently retains more genes and contributes more highly expressed copies than the other (Schnable et al. 2011; Cheng et al. 2016). Such differences in retention and expression are thought to be driven by differences in transposable element composition and epigenetic regulation inherited from divergent parental genomes and stably associated with one subgenome following WGD (Freeling et al. 2015; Bird et al. 2018; Cheng et al. 2018). In an autopolyploid, in contrast, duplicated regions begin as identical copies with no inherited epigenetic regulatory differences, and no mechanism is known that could lead to differential retention over evolutionary time (Garsmeur et al. 2014, but see Yu et al. 2023). The balanced fractionation observed in *C. americana* is consistent with this expectation, and similar patterns have been reported in other autopolyploid systems (Li et al. 2021; Liu and Wang 2023).

These results indicate that diploidization in *C. americana* has proceeded unevenly across levels of genome organization, with extensive structural reorganization contrasting with ongoing symmetric genic diploidization. Resolving remaining uncertainties, including whether segregation patterns are strictly disomic or retain some degree of polysomic inheritance, will require further population genomic analyses.

### Phylogenetic Context of the Most Recent WGD in *C. americana*

*Campanula americana* belongs to a small North American clade of eight species, including *Triodanis*, *Campanula floridana* (=*Rothantella floridana*), and *Campanula reverchonii* (=*Poolea reverchonii*), with the European genus *Legousia* representing its sister lineage (Mansion et al. 2012; Crowl et al. 2016; Morin 2020; Xu et al. 2025). Our phylogenetic analysis indicates that the most recent WGD occurred after the divergence of *Legousia* and before the diversification of this North American clade, suggesting that the duplication is shared rather than unique to *C. americana*. This pattern is consistent with available cytological data, as several taxa of this clade, such as *T. perfoliata* and *T. biflora*, have 28 chromosomes similar to *C. americana* (Gadella 1966). In contrast, there is no evidence of gene duplication in *Legousia* (Gadella 1966; Crowl et al. 2016).

The placement of the WGD before the diversification of this North American clade raises the hypothesis that polyploidy contributed to its early diversification. Sequencing and comparing additional genomes from this group would provide a powerful framework to test for the often cited but poorly supported hypothesis that WGD promotes diversification (Clark and Donoghue 2018). This question is especially relevant given that this clade includes widespread taxa (eg *C. americana*, *T. perfoliata*, *T. biflora*) as well as species restricted to extreme environments (eg *C. reverchonii*, *C. floridana*). If confirmed, this would suggest that shared autopolyploidy may have contributed to both the ecological divergence and diversification of this group.

More broadly, the persistence of duplicated gene content in *C. americana*, despite substantial chromosome-level reorganization, illustrates how the different components of diploidization can progress at different rates (Parisod et al. 2010; Li et al. 2021). Comparative genomic analyses across descendants of this shared WGD could provide a useful framework for examining how duplicated genomes diverge, fractionate, and return toward a diploid-like state.

In conclusion, our chromosome-scale assembly of *C. americana* reveals a signature of three WGD, with the most recent event leading to an autopolyploid genome that has undergone extensive structural rearrangement and gradual gene loss. We show that cytological diploidization appears more advanced than genic diploidization, characterizing *C. americana* as a mesopolyploid at an intermediate stage of diploidization. *Campanula americana* shows no evidence of subgenome dominance or biased fractionation, displaying instead a balanced and symmetric gene loss between homologous regions, consistent with patterns reported in other autopolyploids. These results contribute to a growing body of evidence indicating that fractionation following autopolyploidy proceeds in a largely diffused and symmetric fashion. Beyond clarifying the evolutionary history of *C. americana*, this genome provides a valuable reference for comparative studies across Campanulaceae and for exploring how a shared WGD has shaped genome evolution, diversification, and ecological differentiation within this lineage.

## Materials and Methods

### Plant Material, Sequencing, and Genome Profiling

A single individual of *Campanula americana* was grown under controlled greenhouse conditions at the James Madison University. This individual belongs to the Appalachian genetic lineage, the basal lineage of the species, previously characterized through population genetic studies (Barnard-Kubow et al. 2015). We harvested young leaf tissue, immediately flash-frozen in liquid nitrogen, and shipped to Phase Genomics (Seattle, WA) for DNA extraction and sequencing. High-fidelity long-read sequencing (PacBio HiFi), Hi–C sequencing, as well as genome assembly and scaffolding were performed by Phase Genomics. The PacBio HiFi data was used for genome assembly and preliminary genome profiling, while Hi–C reads supported chromosome-scale scaffolding.

### Genome Assembly and Scaffolding

The *C. americana* genome was assembled using PacBio HiFi long reads in combination with Hi–C sequencing. The assembly was performed with hifiasm (Cheng et al. 2021) using default parameters and incorporated Hi–C data to produce phase-resolved haplotype assemblies. Hi–C data was generated with the Proximo Plant Hi–C 4.0 Kit (Phase Genomics, Seattle, WA), a commercial implementation of the Hi–C protocol (Lieberman-Aiden et al. 2009). Briefly, the chromatin of intact cells was cross-linked with formaldehyde, digested using *Dpn*II, *DdeI*, *Hinf*I, and *Mse*I, and proximity ligated in the presence of biotinylated nucleotides to create chimeric DNA fragments representing physically proximate genomic regions. The resulting molecules were enriched using streptavidin beads and used for the preparation of Illumina-compatible libraries. Libraries were sequenced on an Illumina NovaSeq using paired-end 150 bp reads.

To organize the draft assembly into chromosome-scale scaffolds, Hi–C reads were aligned to the *C. americana* contig assembly and used for Hi–C-based scaffolding. Read mapping was performed using BWA-MEM (Li and Durbin 2010) with the −5SP and -t 8 options for alignment. We marked PCR duplicates with SAMBLASTER (Faust and Hall 2014) and removed non-primary and secondary alignments using samtools (Li et al. 2009) with the -F 2304 flag. The scaffolding was performed with the Phase Genomics' Proximo Hi–C genome scaffolding platform, following the single-phase approach described by Bickhart et al. (Bickhart et al. 2017). This method, similar to LACHESIS (Burton et al. 2013), uses Hi–C contact matrices normalized by the number of restriction sites per contig to generate chromosome-scale scaffolds by optimizing contact frequencies patterns. The scaffolds were manually curated and corrected using Juicebox (Durand et al. 2016).

Finally, to assess chromosome-scale scaffold ordering and orientation, we generated a genome-wide contact matrix using HiCExplorer (Ramírez et al. 2018; Wolff et al. 2022). To do this, we first mapped the Hi–C reads to the final genome assembly using BWA-MEM, followed by the generation of the contact matrix with hicBuildMatrix using a 50-kb bin size and Arima restriction motifs. We filtered low- and extreme-coverage bins using hicCorrectMatrix, and the corrected matrix was visualized with hicPlotMatrix.

### Genome Annotation

We extracted RNA from leaf tissue of the same *C. americana* plant used for PacBio and HiFi sequencing. To better estimate the *C. americana* transcriptomic landscape, we also extracted RNA from flower, leaf, root, and stem tissues of a wild-collected plant from the same lineage (Appalachian). All extractions were performed using the Qiagen RNeasy Plant Mini Kit. We then prepared mRNA-seq libraries and sequenced them on an Illumina NovaSeq platform, generating paired-end 150 bp reads.

To identify and soft mask repetitive elements, RepeatModeler and RepeatMasker were employed using the NCBI repeat database as a reference. The GeneMark-ETP pipeline within BRAKER3 was used to integrate transcriptomic and proteomic evidence for gene prediction. RNAseq data generated in this study was used as transcriptomic evidence. Protein homology evidence was obtained from the OrthoDB (v12), using protein sequences corresponding to the Viridiplantae clade. For AUGUSTUS training and ab initio gene prediction, high-confidence genes identified by GeneMark-ETP were used together with extrinsic evidence-supported hints. Gene predictions from AUGUSTUS and GeneMark-ETP were subsequently integrated using TSEBRA. We filtered the resulting gene set to retain only the longest isoform for each gene. We assigned functional gene names using three complementary annotation sources: homology-based annotation from BLAST searches against the UniProt/Swiss-Prot database, orthology-based annotation using eggNOG, and protein domain annotation using Pfam. Finally, we evaluated the completeness of the annotation using BUSCO (v5.8.3) with the “embryophyta_odb12” lineage dataset.

### Genome Profiling

To obtain a comprehensive overview of the *C. americana* genome composition and complexity, we used k-mer frequency profiling of the HiFi reads to infer genome size, heterozygosity, and ploidy level. We used Jellyfish (Marçais and Kingsford 2011) to generate a 17-mer frequency histogram, counting canonical k-mers (-C). The resulting histogram was analyzed with GenomeScope 2.0 (Ranallo-Benavidez et al. 2020) using parameters *k* = 17 and *P* = 4 to estimate genome size, heterozygosity, and repeat content. Smudgeplot (Ranallo-Benavidez et al. 2020) was used with the same k-mer size (*k* = 17) to infer ploidy structure and assess genome complexity.

### Whole-Genome Duplication Inference Analyses

We used three complementary approaches to infer WGD in *Campanula americana*: *Ks*-based analysis, synteny analysis, and phylogenetic reconciliation. For Ks-based analysis, we modeled the distribution of the synonymous substitution rates (*Ks*) within *C. americana* and between *C. americana* and three other species in the Campanulaceae for which relevant whole-genome assemblies exist: *Codonopsis pilosula*, *Lobelia dortmanna*, and *L. seguinii*. We performed these analyses using WGDI (Sun et al. 2022). Briefly, we carried out BLAST searches within/between genomes and identified collinear blocks using the -icl module in WGDI that detects homologous gene pairs and organizes them into syntenic blocks based on gene order and orientation. WGDI then calculates pairwise *Ks* values between homologous gene pairs within each block, allowing for the construction of *Ks* distributions that reflect either duplication events (intra-genomic comparisons) or divergence (inter-genomic comparisons). We estimated the timing of WGD events in *C. americana* based on the Ks values of each peak in the Ks distribution using the formula *T* = *Ks/2µ* assuming a synonymous mutation rate (*µ*) of 6.1 × 10^−9^ substitutions/site/year (Wolfe et al. 1989; Gaut 1998; Gaut et al. 2011).

For synteny-based inference, we used the collinearity block data from WGDI to estimate synteny depth. We projected syntenic blocks of the *C. americana* genome onto each of the three other Campanulaceae species, overlaid all blocks, and divided the genome into continuous segments. Each segment was assigned a depth equal to the number of overlapping blocks. A length-weighted histogram was generated, and the modal depth (covering the largest genome fraction) was used to infer the number of WGD events. For example, a modal depth of ∼2 suggests one WGD, while ∼4 suggests two WGD events.

We used phylogenetic reconciliation at two taxonomic scales to investigate the placement of the WGD associated with *C. americana*. First, we used genome-wide sequence data from several Campanulaceae *s.l.* and more distantly related taxa to place the WGD in a broad phylogenetic context. We then performed a second analysis with denser sampling of close relatives of *C. americana* to provide a refined placement of the duplication.

To place WGDs in a broad phylogenetic context, we retrieved genomic or transcriptomic data from multiple Campanulaceae, members of the closely related family Asteraceae, and *Cornus florida* and *Camellia sinensis* as outgroups (Table S6). For species with only RNA-seq data, we performed de novo transcriptome assembly using Trans-ABySS (Robertson et al. 2010) with *k*-mer lengths of 32, 48, 64, and 72 and merging assemblies with transabyss-merge. We used CD-HIT (Huang et al. 2010) with -c 0.95 and -aS 0.9 to retain the longest isoform per gene. We then predicted protein sequences using TransDecoder, retaining those with BLAST hits to the UniProt database. For species with annotated genomes, coding and protein sequences of the longest isoform per gene were extracted from the genome annotations. Homologous genes across species were identified using OrthoFinder. The sequences of each orthogroup were aligned with MAFFT v7.505 (Katoh and Standley 2013), and Gblocks was used to filter out gappy sites and highly divergent regions. Orthogroups that resulted in alignments with zero nucleotides (∼2,000) were excluded, leaving a total of 4,900 orthogroups. To infer WGD via phylogenetic reconciliation, we first constructed a species tree using ASTRAL-III v5.7.8 (Zhang et al. 2018) based on all 4,900 orthogroups. We then used Tree2GD (Chen et al. 2022) to identify nodes with a high proportion of gene duplications (>10%), which were interpreted as evidence of WGD events within the corresponding clades.

To refine the placement of the WGD near *C. americana*, we assembled 659 single-copy nuclear genes from 18 Campanulaceae species using HybPiper v2.2.0 (Johnson et al. 2016), including publicly available short-read data from 17 species (Xu et al. 2025) and *C. americana* data generated for this study (Table S7). The target 659 nuclear genes were recently identified in the transcriptome of *Adenophora polyantha* (Xu et al. 2025). We aligned each gene using MAFFT (--auto) and filtered them to retain loci with ≥70% taxon occupancy. We trimmed the alignments with trimAl using -gt 0.8 and -st 0.001 (Capella-Gutiérrez et al. 2009) and inferred gene trees with IQ-TREE 3 (Wong et al. 2026), collapsing branches with bootstrap support ≤10. The 510 retained gene trees were used to estimate the species tree with ASTRAL-III. Then, we identified paralogous sequences with HybPiper's paralog_retriever.py script. Paralogous gene trees were processed using the same alignment and occupancy filters and used to infer gene trees using IQ-TREE. The 112 resulting trees were reconciled against the ASTRAL species tree using Tree2GD to identify branches with elevated duplication. Because duplication events were concentrated on two adjacent branches surrounding the divergence of *Legousia* and the focal *Campanula–Triodanis* clade, we inspected the supporting gene trees to evaluate the relative placement of the WGD. All phylogenetic trees were visualized using iTOL v6 (Letunic and Bork 2024).

### Phylogenetic Assessment of Polyploid Origin

To assess the mode of the WGD in *C. americana*, we examined all filtered paralog gene trees (112 trees) used for phylogenetic reconciliation containing at least two *C. americana* copies (69 trees). First, trees were rooted with *C. persicifolia* or *C. stevenii*, and copies were classified as grouping by species when the most recent common ancestor of the *C. americana* copies included no other taxa (Fig. 3 upper left panel) or by paralog lineage when each copy fell in a distinct clade containing other *Campanula–Triodanis* species (Fig. 3 upper central and right panel). Second, we identified the closest external lineage of each copy in the subset of trees in which the rooting taxa, *C. persicifolia* and *C. stevenii*, formed a monophyletic group (54 trees). Then, for each *C. americana* copy, we recorded the first external lineage encountered toward the root, excluding other members of the focal *Campanula–Triodanis* clade. We considered an external phylogenetic relationship supported only when the relevant node had a bootstrap support of ≥50. We then compared the external lineages of duplicated *C. americana* copies within each gene: association with different external lineages would suggest parents from divergent lineages, whereas association with the same lineage would exclude that scenario.

### Subgenome Inference

To evaluate potential chromosome-scale subgenome differentiation, we analyzed the *C. americana* genome using SubPhaser. First, we identified collinear blocks between a chromosome pair associated with the most recent WGD using whole-genome dotplot comparisons. Ten collinear blocks with median *Ks* between 0.02 and 0.25 were used as input for the SubPhaser analysis. In addition, we used collinear blocks from all chromosomes as input to evaluate whether subgenome assignments were consistent at the whole-genome scale. We ran SubPhaser using default parameters while varying the minimum k-mer frequency threshold (-q). We conducted analysis with *q* = 50, 25, 17, and 12, such that only k-mers occurring at least *q* times were retained for subgenome assignment. Chromosome partitioning was evaluated using differential k-mer enrichment, hierarchical clustering of k-mer abundance, and comparison of LTR-RT insertion age profiles between inferred groups.

To assess whether sequence divergence varied among chromosome pairs, we analyzed *Ks* among collinear anchor gene pairs associated with the most recent WGD identified with WGDI. Collinear blocks were obtained from whole-genome comparisons, and gene pairs within each block were used to extract pairwise *Ks* estimates. *Ks* values were filtered to remove invalid or extreme estimates (*Ks* < 0 or *Ks* > 2). Pairwise *Ks* distributions were summarized by chromosome pair and compared to assess heterogeneity in divergence across the genome.

### Genic Evolution in Campanula americana

We explored the patterns of retention/loss of gene duplicates and whether subgenome-like dominance is found between homologous regions of *C. americana*. As shown above, *C. americana* underwent three WGD events. However, for these analyses, we focused on gene retention following the most recent WGD since our genome profiling indicated a strong tetraploid architecture (see Results section).

To ascertain subgenome dominance in a polypoid species, a closely related species that has not undergone WGD is typically used as reference for comparison of gene retention patterns between homologs. However, no such genome exists relative to *C. americana*. Therefore, we constructed a “consensus chromosomal block” using the within-genome synteny information. Specifically, for each syntenic block identified between homologous regions, we defined a consensus chromosomal block as the union of all genes from both homologs. Each consensus gene was then coded as present or absent in each homolog, resulting in a binary presence/absence matrix. Using this matrix, we quantified genome-wide retention asymmetry across all collinear blocks. Since chromosomes could not be assigned into subgenomes, we measured retention asymmetry as the absolute difference in retention between the two chromosomes forming each block. For each block, the proportion of retained genes was calculated independently for each homolog, and the asymmetry was calculated as |Δ_retention_| = |*p_A_–p_B_*|, where *p_A_* and *p_B_* represent the proportion of retained genes in each homolog. This metric quantifies the magnitude of retention without assuming a subgenome structure. The genome-wide retention asymmetry was obtained by calculating the mean, standard deviation, and distribution of |Δ_retention_| across all collinear blocks. Genes originated by tandem duplications, likely reflecting post-WGD events, were identified using DupGen_Finder (Qiao et al. 2019) and excluded from the analysis after the construction of the consensus chromosomal blocks.

Finally, using the same presence/absence matrix, we investigated the fine-scale pattern of gene loss across homologous regions. Specifically, we asked whether gene loss occurred as isolated events or in contiguous segments. To assess this, we calculated the lengths of consecutive runs of gene loss within each relative to the consensus and plotted their frequency distribution across all collinear blocks.

## Supplementary Material

evag252_Supplementary_Data

## Data Availability

The sequencing PacBio HiFi, HiC, RNAseq, and Illumina short-read data underlying this article are available at NCBI in the SRA repository under BioProject PRJNA1363131. The annotated genome assemblies (Haplotype 1 and Haplotype2) are available at NCBI under BioProjects PRJNA1365259 and PRJNA1365260. The scripts used in this work and the paralog gene trees used to explore the autopolyploid origin of *C. americana* are available at https://github.com/Alfredo-LC/Camericana_ReferenceGenome.
