# Supplementary material for: Genomic Signatures of Polyploid Origin and Diploidization in Campanula americana
Source: Genome Biol Evol. 2026 Oct 14;18(10):evag252. doi: 10.1093/gbe/evag252 (PMC13696962; doi:10.1093/gbe/evag252)
Supplement: Supplementary file 1 [file evag252_supplementary_data.docx]

**Figure S1**. Genome wide Hi-C contact map of the *Campanula americana* haplotype 1. The interaction frequencies were plotted across the assembly using 50-Kb bins. Chromosomes are ordered along both axes, and contact frequency is shown in a log-transformed scale. The map shows strong diagonal signal and low-off diagonal interaction, supporting the ordering and orientation of scaffolds in the final genome assembly.

**Figure S2**. ASTRAL species tree of the *Campanula americana* close relatives based on 510 single-copy genes. All nodes have LPP > 0.9 except nodes with an asterisk. Tree2GD was used to conduct phylogenetic reconciliation. We show nodes with GDratio > 5%, suggesting the occurrence of a WGD event. Two nodes with elevated WGD duplication signal occur near *Campanula americana*.

**Figure S3**. Ks distribution among homologous chromosome pairs in *Campanula americana*. Pairwise Ks values were calculated for collinear gene pairs associated with the most recent WGD inferred with WGDI and summarized by chromosome pair. Across all chromosome pairs, Ks distributions show similar medians and broadly overlapping ranges. This genome-wide similarity is consistent with an autopolyploid origin of *C. americana*, rather than divergence among distinct parental subgenomes expected under allopolyploidy.

**Supplemental Table 1**. Genome sequencing data of *Campanula americana*

| Sequencing | *C. americana* |
| --- | --- |
| PacBio HiFi sequencing |  |
| Number of reads | 7,389,887 |
| Raw data | 82.16 Gb |
| Mean read length (bp) | 11,117 bp |
| Median read quality | 37 |
| Hi-C sequencing |  |
| Number of read pairs | 691,708,242 |

**Supplemental Table 2**. Hi-C assembly data for the *C. americana* genome

| Item | Haplotype 1 | Haplotype 2 |
| --- | --- | --- |
| No. of scaffold | 29 | 29 |
| Length of scaffolds | 1.69 Gbp | 1.30 Gbp |
| No. of contigs in scaffolds | 193 | 182 |
| % of contigs in scaffolds | 86% | 98% |
| Unscaffolded contigs | 3078 | 891 |
| N50 | 57.55 Mb | 50 Mb |
| No. of genes | 46,149 | 42,887 |
| BUSCO completeness | 99.60% | 98.80% |

**Supplemental Table 3**. Pseudochromosome length in haplotype 1 and haplotype 2 for *C. americana*

| **Pseudochromosome** | **Haplotype 1 length (bp)** | **Haplotype 2 length (bp)** |
| --- | --- | --- |
| Chr01 | 73,879,077 | 62,843,353 |
| Chr02 | 57,332,316 | 58,035,056 |
| Chr03 | 67,789,591 | 57,334,864 |
| Chr04 | 63,539,111 | 56,728,725 |
| Chr05 | 59,204,642 | 56,563,362 |
| Chr06 | 68,501,741 | 54,641,702 |
| Chr07 | 66,763,199 | 54,407,849 |
| Chr08 | 59,032,497 | 53,878,157 |
| Chr09 | 53,222,376 | 52,734,428 |
| Chr10 | 51,841,740 | 50,792,221 |
| Chr11 | 58,452,759 | 50,718,847 |
| Chr12 | 57,874,618 | 50,159,653 |
| Chr13 | 51,016,350 | 50,092,791 |
| Chr14 | 54,409,421 | 48,376,348 |
| Chr15 | 49,307,962 | 46,394,494 |
| Chr16 | 58,193,923 | 44,720,230 |
| Chr17 | 49,771,500 | 44,533,061 |
| Chr18 | 57,551,242 | 43,592,435 |
| Chr19 | 53,589,107 | 40,782,783 |
| Chr20 | 68,029,791 | 40,760,129 |
| Chr21 | 64,134,622 | 40,560,064 |
| Chr22 | 40,022,855 | 38,828,055 |
| Chr23 | 73,619,945 | 38,244,909 |
| Chr24 | 48,922,678 | 35,441,099 |
| Chr25 | 59,972,646 | 25,357,827 |
| Chr26 | 56,361,864 | 24,976,209 |
| Chr27 | 63,893,407 | 45,277,999 |
| Chr28 | 54,772,996 | 20,029,296 |
| Chr29 | 55,525,400 | 18,794,540 |

**Supplemental Table 4**. BUSCO completeness assessment of the genome assemblies and of the gene prediction of *C. americana* based on the 2026 genes of the embryophyta_odb12 database.

| BUSCO classification | Haplotype 1 | | Haplotype 2 | |
| --- | --- | --- | --- | --- |
|  | Genome assembly | Gene prediction | Genome assembly | Gene prediction |
| Complete | 98.5% | 97.7% | 97.1% | 87.8% |
| Complete and single-copy | 25.8% | 33.3% | 28.9% | 14.1% |
| Complete and duplicated | 72.7% | 64.4% | 68.2% | 73.7% |
| Fragmented | 1.0% | 0.8% | 1.3% | 1.6% |
| Missing | 0.5% | 1.5% | 1.6% | 10.6% |

**Supplemental Table 5**. Repetitive elements in the *C. americana* genome

| Type | Number of elements | Length (bp) | Percentage of sequence |
| --- | --- | --- | --- |
| Retroelements | 511,079 | 870,947,485 | 44.14 |
| SINEs | 7,445 | 2,556,279 | 0.13 |
| LINEs | 41,644 | 31,127,161 | 1.58 |
| Penelope | 532 | 135,679 | 0.01 |
| *Copia* | 77,167 | 134,319,940 | 6.81 |
| *Gypsy* | 367,852 | 694,643,692 | 35.21 |
| DNA transposons | 81,811 | 56,436,110 | 2.86 |
| Unclassified | 1,222,028 | 525,004,564 | 26.61 |
| **Total interspersed repeats** | **1,798,479** | **1,452,388,159** | **73.61** |
| Small RNA | 19,840 | 31,602,914 | 1.6 |
| Satellites | 21,323 | 10,259,633 | 0.52 |
| **Total repetitive elements** | **1,839,642** | **1,494,250,706** | **75.73** |

**Supplemental Table 6**. Species used in this study for whole-genome duplication inference through phylogenetic, Ks-based and synteny analysis

| **Species** | **Family** | **Data Type** | **Link** |
| --- | --- | --- | --- |
| *Cornus florida* | Cornaceae | Genome annotation | <https://www.ncbi.nlm.nih.gov/datasets/genome/GCF_030987335.1/> |
| *Camellia sinensis* | Theaceae | Genome annotation | <https://www.ncbi.nlm.nih.gov/datasets/genome/GCF_004153795.1/> |
| *Cichorium intybus* | Asteraceae | Genome annotation | <https://www.ncbi.nlm.nih.gov/datasets/genome/GCA_023525715.1/> |
| *Cynara cardunculus* | Asteraceae | Genome annotation | <https://www.ncbi.nlm.nih.gov/datasets/genome/GCF_001531365.2/> |
| *Erigeron canadensis* | Asteraceae | Genome annotation | <https://www.ncbi.nlm.nih.gov/datasets/genome/GCF_010389155.1/> |
| *Helianthus annuus* | Asteraceae | Genome annotation | <https://www.ncbi.nlm.nih.gov/datasets/genome/GCF_002127325.2/> |
| *Smallanthus sonchifolius* | Asteraceae | Genome annotation | <https://www.ncbi.nlm.nih.gov/datasets/taxonomy/185202/> |
| *Lobelia aberdarica* | Campanulaceae | raw RNAseq data | <https://www.ncbi.nlm.nih.gov/sra/?term=SRR3180742> |
| *Lobelia cardinalis* | Campanulaceae | raw RNAseq data | <https://www.ncbi.nlm.nih.gov/sra/?term=SRR12034797> |
| *Lobelia dortmanna* | Campanulaceae | Genome assembly | <https://www.ncbi.nlm.nih.gov/datasets/genome/GCA_965199305.1/> |
| *Lobelia seguinii* | Campanulaceae | Genome annotation | <https://doi.org/10.6084/m9.figshare.26860564> |
| *Lobelia telekii* | Campanulaceae | raw RNAseq data | <https://www.ncbi.nlm.nih.gov/sra/?term=SRR3180743> |
| *Platycodon grandiflorus* | Campanulaceae | Genome annotation | <https://www.ncbi.nlm.nih.gov/datasets/genome/GCA_016624345.1/> |
| *Codonopsis lanceolata* | Campanulaceae | Genome annotation | [https://ngdc.cncb.ac.cn/gwh/Assembly/85966/show, GWHFAJJ00000000.1, https://doi.org/10.6084/m9.figshare.26799025](https://ngdc.cncb.ac.cn/gwh/Assembly/85966/show,%20GWHFAJJ00000000.1) |
| *Nesocodon mauritianus* | Campanulaceae | raw RNAseq data | <https://www.ncbi.nlm.nih.gov/sra/?term=SRR11688926> |
| *Campanula americana* | Campanulaceae | Genome annotation | This study |
| *Campanula cochleariifolia* | Campanulaceae | raw RNAseq data | <https://www.ncbi.nlm.nih.gov/sra/?term=SRR12002082> |
| *Campanula takesimana* | Campanulaceae | raw RNAseq data | <https://www.ncbi.nlm.nih.gov/sra/?term=SRR11804864> |
| *Adenophora polyantha* | Campanulaceae | raw RNAseq data | <https://www.ncbi.nlm.nih.gov/sra/?term=SRR11994226> |
| *Adenophora stricta* | Campanulaceae | raw RNAseq data | <https://www.ncbi.nlm.nih.gov/sra/?term=SRR31592529> |
| *Adenophorta triphylla* | Campanulaceae | raw RNAseq data | <https://www.ncbi.nlm.nih.gov/sra/?term=SRR26045271> |

**Supplemental Table 7**. Species and SRA accession numbers of the short-read sequencing data used for phylogenetic reconciliation within Campanulaceae

| **Species** | **ID** |
| --- | --- |
| *Asyneuma chinense* | SRR22197999 |
| *Asyneuma japonicum* | SRR22197997 |
| *Asyneuma thomsonii* | SRR24743424 |
| *Campanula americana* | pending |
| *Campanula floridana* | SRR24743423 |
| *Campanula persicifolia* | SRR22197950 |
| *Campanula reverchonii* | SRR24743420 |
| *Campanula stevenii* | SRR22197994 |
| *Campanula uniflora* | SRR24743422 |
| *Legousia hybrida* | SRR22197921 |
| *Legousia speculum-veneris* | SRR22197920 |
| *Petromarula pinnata* | SRR22197916 |
| *Phyteuma orbiculare* | SRR22197915 |
| *Phyteuma spicatum* | SRR22197914 |
| *Triodanis coloradoensis* | SRR24743419 |
| *Triodanis lamprosmerma* | SRR24743425 |
| *Triodanis biflora* | SRR22197978 |
| *Triodanis perfoliata* | SRR22197977 |
